# Synthesis and anticancer evaluation of novel 3,5-diaryl-thiazolo[4,5-*d*]pyrimidin-2-one derivatives

**DOI:** 10.1007/s00044-012-0231-7

**Published:** 2012-10-04

**Authors:** Lilianna Becan, Edwin Wagner

**Affiliations:** Department of Drugs Technology, Wroclaw Medical University, Pl. Nankiera 1, 50-140 Wroclaw, Poland

**Keywords:** Antitumor agents, In vitro studies, Thiazolo[4,5-*d*]pyrimidin-2-ones, Synthesis

## Abstract

The 2-oxo analogs of thiazolo[4,5-*d*]pyrimidine-2-thiones were prepared to study their cytotoxic activity. Five of the newly synthesized compounds were selected by the National Cancer Institute (Bethesda, MD, USA) for a primary in vitro antitumor assay. 7-Chloro-3,5-diphenyl-thiazolo[4,5-*d*]pyrimidin-2-one (**5a**) proved to be the most active one among the screened derivatives and was further evaluated in the full panel of 60 cell lines at five different concentrations. The structures of compounds were determined by IR, ^1^H-NMR, ^13^C-NMR, X-ray, and elemental analysis.

## Introduction

Thiazolo[4,5-*d*]pyrimidines, 7-thio analogs of purines are potentially bioactive molecules. In contrast with related 2-thioxo-thiazolo[4,5-*d*]pyrimidine derivatives, the 2-oxo analogs have not been very well explored in medicinal chemistry. The synthesis and biological evaluation of the substituted 2-oxo-thiazolo[4,5-*d*]pyrimidines have been the subject of several review articles. They were reported to possess antibacterial, antifungal (Akbari *et al*., 2008; Habib *et al*., 2007), and anti-inflammatory activity (CXCR2-receptor antagonists) (Walters *et al*., 2008), inhibit the growth of HCMV-human cytomegalovirus (Revankar *et al*., 1998), and be corticotrophin-releasing hormone (CRH-R1) receptor antagonists (display antidepressant activity) (Beck *et al*., 1999).

**Fig. 1:**
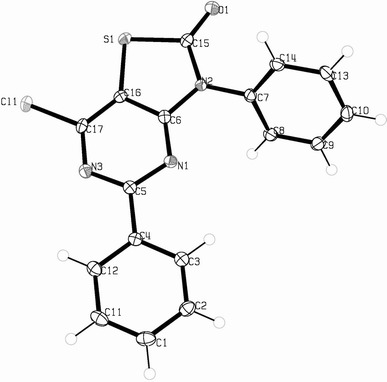
X-Ray molecular structure of compound **5a** with the atom-numbering scheme used in the crystallographic analysis

**Scheme 1:**
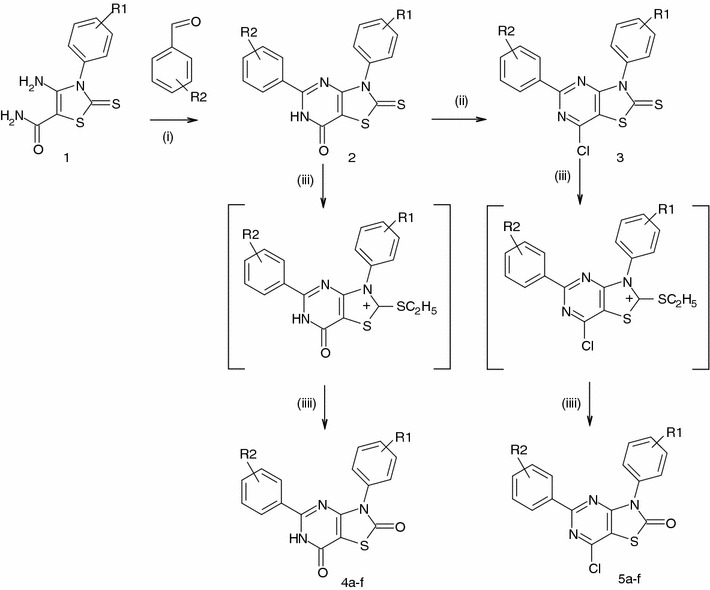
(i) LiOH; (ii) POCl_3_/PCl_5_; (iii) (C_2_H_5_)_2_SO_4_; (iiii) H_2_O

In this study, in continuation of our work on thiazolo[4,5-*d*]pyrimidine derivatives, the synthesis and in vitro cytotoxic evaluation of thiazolo[4,5-*d*]pyrimidin-2-ones are reported. These designed thiazolo[4,5-*d*]pyrimidine-2-ones are related to thiazolo[4,5-*d*]pyrimidine-2-thiones that have been previously reported to be potent antitumor agents (Becan and Wagner, 2008). Thiazolo[4,5-*d*]pyrimidine derivatives have been extensively studied as potential drug candidates and also have anticancer activity (Rida *et al*., 1996; Fahmy *et al*., 2002, 2003). Most of these compounds provided with anticancer activity possess an aromatic rings and electronegative substituent directly linked to the C-17 of the essential core (Fig. 1) or attached at aromatic moieties. The method involved subsequent treatment of the appropriate 3,5-diaryl-2-thioxo-5,6-dihydro-4*H*-thiazolo[4,5-*d*]pyrimidin-7-ones (**2**) and 7-chloro-3,5-diaryl-thiazolo[4,5-*d*]pyrimidine-2-thiones (**3**) with diethyl sulfate and water for the replacement of the 2-thioxo group by an oxygen function (Scheme 1). Compounds **2** and **3** were obtained from 4-amino-5-carboxamido-3-substituted-2,3-dihydrothiazole-2-thiones (**1**) (Gewald, 1966) according to a reported earlier procedure (Becan and Wagner, 2008). Pyrimidine ring formation with appropriate aryl aldehyde, followed by chlorination provided the desired cores **2** and **3**, bearing the respective aromatic substituent at position 3 and 5, which could further be treated with diethyl sulfate and hydrolyzed to yield 2-thiazolones **4a**–**4f** and **5a**–**5f**. All synthesized compounds were submitted to the National Cancer Institute (NCI, Bethesda, Maryland) to evaluate their growth inhibitory effects on 60 human cancer cell lines, derived from nine neoplasmatic diseases. Five derivatives **4a**, **4b**, **5a**, **5b**, and **5d** were selected for a primary in vitro antitumor assay, at 10^−5^ M concentration. Results were expressed as percent growth of the treated cells, compound **5a** showing mean percent growth =71.26 was further tested at five different concentrations.

## Results and discussion

### Chemistry

A series of the new substituted thiazolo[4,5-*d*]pyrimidin-2-ones **4a**–**4f** and **5a**–**5f** were synthesized as shown in Scheme 1. The method involved subsequent treatment of the appropriate 3,5-diaryl-2-thioxo-5,6-dihydro-4*H*-thiazolo[4,5-*d*]pyrimidin-7-ones (**2**) and 7-chloro-3,5-diaryl-thiazolo[4,5-*d*]pyrimidine-2-thiones (**3**) (Becan and Wagner, 2008) with diethyl sulfate and water for the replacement of the 2-thioxo group with 2-oxo. First, compounds **2** were obtained through the reaction of the corresponding, refluxing aromatic aldehyde with 4-amino-5-carboxamido-3-substituted-2,3-dihydrothiazole-2-thiones **1** (Gewald, 1966), in the presence of bases according to the earlier reported procedure (Becan and Wagner, 2008). Pyrimidine ring formation with aryl aldehydes followed by chlorination with a mixture of phosphorus pentachloride and phosphorus oxychloride gave the desired cores **2** and **3** which were further treated in boiling acetonitrile with diethyl sulfate. The obtained positively charged 2-ethyltiothiazolium salt was hydrolyzed to yield thiazolones-2. Yields of reaction were variable and were higher when R1 and R2 were not substituted. Elemental analysis, IR, ^1^H and ^13^C-NMR, and X-ray data evaluated the structure of synthesized substances. In the IR spectra of compounds **4a**–**4f**, the two stretching bands of 6-NH group were detected in the range of 3470–3080 cm^−1^. These compounds showed the characteristic vibrations of the C=O group at 1690–1670 cm^−1^. In the ^1^H-NMR spectra, characteristic signal of compounds **4a**–**4f** was one-proton singlet of 6 N–H resonated at 13.19–13.27 ppm. Aromatic protons have formed multiplet at 7.22–8.20 ppm.

The formation of chlorination products **5a**–**5f** was indicated in the IR spectra by the disappearance of stretching bands of 6-NH group. Besides the absorption bands due to C=N and C–S–C functions, the presence of C=O functional group was marked by the appearance of bond ranging from 1690 to 1680 cm^−1^, which was lacked in the precursor **3**. In the ^1^H-NMR spectra of 7-chloro derivatives **5a**–**5f** we were observed only aromatic protons signal at 7.26–8.22 ppm. The ^13^C-NMR spectra of the active compounds **5a**, **5b**, and **5d**, given in Table 1, displayed the appropriate number of resonances that exactly fit the number of carbon atoms. The most active compound **5a** was recrystallized from a DMF solvent; the block-shaped crystals formed as a result were submitted to X-ray analysis. Data were collected at 100 K from a single crystal. X-ray crystallography of the most active agent **5a** confirmed the chemical structure (Fig. 1). Crystallographic data for the structure are depicted in Table 2.

**Table 1:** ^13^C-NMR data of compounds **5a**, **5b**, and **5d**

Comp.	^13^C-NMR (DMSO-d_6_) δ ppm

**5a** R2=H	168.25 (C15), 166.79 (C5), 160.99 (C7), 157.65 (C17), 150.71 (C4), 135.08 (C6), 133.55 (C16), 133.17 (C1), 132.04 (C10), 131.69 (C13), 129.44 (C9), 129.28 (C11), 129.04 (C2), 128.94 (C3), 128.86 (C12), 128.70 (C14), 128.05 (C8)
**5b** R2=Cl	168.21 (C15), 166.73 (C5), 159.96 (C17), 157.67 (C7), 155.87 (C4), 150.71 (C6), 136.87 (C16), 136.54 (C1), 133.96 (C10), 133.52 (C3), 133.11 (C12), 130.66 (C13), 129.34 (C9), 129.07 (C14), 129.03 (C8), 128.93 (C11), 128.81 (C2)
**5d** R2=F	168.21 (C15), 166.75 (C5), 160.04 (C1), 157.59 (C17), 155.64 (C7), 150.71 (C4), 133.49 (C6), 133.11 (C16), 131.60 (C10), 130.50 (C3), 130.38 (C12), 130.19 (C9), 130.07 (C14), 129.16 (C8), 129.30 (C13), 115.97 (C2), 115.76 (C11)

The carbon atom-numbering scheme used in the crystallographic analysis was applied

**Table 2:** Crystallographic data for compound **5a**

Crystal data and structure refinement	
Empirical formula	C_17_H_10_ClN_3_O_2_S
Formula weight	339.79
Temperature	100(2) K
Wavelength	0.71073 Å
Crystal system, space group	Monoclinic, Cc
Unit cell dimensions	*a* = 11.7588 (8) Å α = 90˚
	*b* = 19.4837 (14) Å β = 90˚
	*c* = 7.0758 (5) Å γ = 90˚
Volume	1468.89 (18) Å^3^
Z, calculated density	4, 1.536 Mg/m^3^
Absorption coefficient	0.409 mm^−1^
F (000)	696
Crystal size	0.20 × 0.10 × 0.10 mm
Theta range for data collection	2.18–27.07˚
Limiting indices	−15 ⇐ h ⇐ 15, −24 ⇐ k ⇐ 24, −9 ⇐ l ⇐ 9
Reflection collected/unique	61,281/3,225 [*R* (int) = 0.0320]
Completeness to theta = 27.07	99.9 %
Absorption correction	Semi-empirical from equivalents
Max. and min transmission	0.9602 and 0.9226
Refinement method	Full-matrix least-squares on *F* ^2^
Data/restraints/parameters	3,225/3/208
Goodness-of-fit on *F* ^2^	1.036
Final R indices [*I* > 2sigma (I)]	*R*_1_ = 0.0195, *wR*_2_ = 0.0520
R indices (all data)	*R*_1_ = 0.0197, *w*R_2_ = 0.0524
Absolute structure parameter	−0.02 (3)
Largest diff. peak and hole	0.202 and −0.265 e.Å^3^

### Anticancer activity assay

All synthesized compounds were submitted for testing at the NCI to evaluate the growth inhibitory effect. Five compounds **4a**, **4b**, **5a**, **5b**, and **5d** were selected for a primary in vitro antitumor assay (Monks *et al*., 1991; Boyd and Paull, 1995; Shoemaker *et al*., 2002). A process beginning with the evaluation of the compound against approximately 60 different human tumor cell lines representing leukemia, melanoma, and cancers of the lung, colon, brain, breast, ovary, prostate, and kidney at 10^−5^ M concentration was performed. With one dose, compound **4b** was devoid of cytotoxic activity (mean growth percent 99.88) and **4a** was slightly active against renal cancer CAKI-1 cell line (26.76 % growth).

Compounds **5a**, **5b**, and **5d** which possess electron-withdrawing 7-chloro substituent showed variable antitumor activity, reported as the percentage of growth of treated cells; the preliminary screening results are shown in Table 3. Compounds **5a**, **5b**, and **5d** exhibited antiproliferative effect against cell lines of leukemia, non-small cell lung cancer, colon cancer, melanoma, ovarian cancer, and renal cancer. It is worth to mention that substance **5a** showed noticeable cytotoxic activity against renal cancer (UO-31), melanoma (MALME-3M), and non-small cell lung cancer (NCI-H522) while compound **5b** was most effective against the last one. This limited data indicate that the replacement of the 7-oxo group with the small, non-polar chloro substituent substantially increased anticancer activity. Remarkable low growth percent values against a minimum number of cell lines (mean growth) was obtained only for compound **5a** which was approved for the further screening test to evaluate the growth inhibition (GI), and cytostatic and cytotoxic effects. The selected compound was additionally evaluated at tenfold dilution of five different concentrations, from 10^−4^ to 10^−8^ M on approximately 60 human tumor cell lines panels. Three different dose–response parameters, GI_50_, TGI, and LC_50_, were calculated for each cell line. GI_50_ is the molar concentration of the compound required for half GI. Total growth inhibition (TGI) is the molar concentration of the compound resulting in TGI; TGI signifies the cytostatic effect. LC_50_ is the molar concentration of the compound resulting in a 50 % death of the initial cells; LC_50_ signifies the cytotoxic effect. The overview of these parameters of compound **5a** is reported in Table 4 and compared with log GI_50_ values of thioguanine (TG), the NCI standard anticancer agent. The log GI_50_ values lower than −5 showed a notable activity level. It can be noticed that compound **5a** proved to be very sensitive toward non-small cell lung cancer NCI-H522 and renal cancer UO-31 log GI_50_ −5.91 and −5.88, respectively, (MG_MID: log GI_50_ −5.1, log TGI −4.4, log LC_50_ −4.09). GI of most cell lines of standard TG is higher than that showed by investigated compound **5a**; but against the following cell lines: K-562, NCI-H322M, NCI-H522, SW-620, U251, SK-MEL-28, IGROV1, A498, and HS 578T, compound **5a** was more active than TG. TG is a guanine analog and thiazolo[4,5-*d*]pyrimidines can be considered as 7-thio analogs of the purine bases guanine and adenine. Thiazolo[4,5-*d*]pyrimidine derivatives may interfere with the synthesis of guanine nucleotides as antimetabolites.

**Table 3:** Anticancer activity as growth % in concentration 10^−5^ M for the compounds **5a**, **5b**, and **5d**

Compound	Mean growth%	Range of growth%	Most sensitive panel/cell line growth *%*
**5a**	71.26	−84.63 to 124.07	−84.63 Rc/UO-31, −77.98 M/MALME-3M, −69.53 NSCLc/NCI-H522, 3.17 Cc/HCC-2998, 8.46 Cc/HCC-116, 16.05 M/LOX IMVI, 19.57 L/CCRF-CEM, 26.33 L/SR, 33.32 Oc/OVCAR-3
**5b**	86.17	5.19 to 136.81	5.19 NSCLc/NCI-H522, 21.51 L/SR,24.35 M/LOX IMVI, 29.34 Cc/HCT-116, 33.63 L/CCRF-CEM, 34.56 L/K-562, 47.57 Cc/SW-620
**5d**	91.21	−31.63 to 124.32	−31.63 NSCLc/NCI-H522, 28.57 L/SR, 35.79 L/K-562, 40.46 Cc/HCT-116, 41.76 Rc/CAKI-1

Data obtained from the NCIs in vitro disease-oriented human tumor cells

*L* leukemia, *NSCLc* non-small cell lung cancer, *Cc* colon cancer, *M* melanoma, *Oc* ovarian cancer, *Rc* renal cancer

**Table 4:** The result of the in vitro anticancer activity of compound **5a** against 60 human cancer cell lines

Panel Cell line	Compound **5a**				TG
	Growth (%)^b^ 10^−5^ M	Log Gl_50_	Log TGI	Log LC_50_	Log Gl_50_
*Leukemia*					
CCRF-CEM	23	−5.42	–^c^	–	−6.5
HL-60(TB)	38	−5.23	–	–	ns
K-562	19	−5.39	–	–	−4.0
MOLT-4	54	−4.89	–	–	ns^d^
RPMI-8226	75	−4.35	–	–	−5.9
SR	8	−5.58	−4.67	–	−6.2
*Non*-*Small*					
*Cell Lung C.*					
A549/ATCC	87	−4.36	–	–	−5.3
EKVX	84	−4.59	–	–	−5.4
HOP-62	15	−5.44	−4.81	−4.14	−6.1
HOP-92	24	−5.51	−4.17	–	−5.8
NCI-H23	20	−5.44	−4.51	–	−5.5
NCI-H322 M	62	−4.85	−4.23	–	−4.6
NCI-H460	78	−4.60	–	–	−6.0
NCI-H522	−65	−5.91	−5.52	–	−5.7
*Colon C.*					
COLO-205	52	−4.95	–	–	−5.6
HCC-2998	90	−4.09	–	–	ns
HCT-116	−53	−5.68	−5.35	−5.02	−6.2
HCT-15	28	−5.33	–	–	−5.6
HT29	10	−5.41	−4.72	–	−5.9
KM12	81	−4.09	–	–	−5.5
SW620	−4	−5.56	−5.04	–	−5.4
*CNS Cancer*					
SF-268	52	−4.98	−4.42	–	−5.9
SF-295	92	−4.24	–	–	−5.9
SF-539	52	−4.96	–	–	−6.2
SNB-19	70	−4.38	–	–	−4.1
SNB-75	12	−5.73	−4.86	−4.25	−6.0
U251	20	−5.43	−4.73	–	−5.0
*Melanoma*					
LOX IMVI	−44	−5.69	−5.32	−4.74	ns
MALME-3M	62	−4.83	−4.10	–	−5.5
M14	16	−5.42	−4.45	–	−6.2
MDA-MB-435	26	−5.31	−4.34	–	−6.3
SK-MEL-2	48	−5.04	−4.36	–	−5.8
SK-MEL-28	9	−5.47	−4.88	−4.16	−5.2
SK-MEL-5	60	−4.81	–	–	−5.6
UACC-257	48	−5.05	−4.50	–	−5.2
UACC-62	62	−4.70	–	–	−6.4
*Ovarian C.*					
IGROV1	−65	−5.75	−5.32	−4.74	−5.2
OVCAR-3	−41	−5.75	−4.10	–	−5.8
OVCAR-4	31	−5.30	−4.45	–	−5.3
OVCAR-5	90	–	−4.34	–	−6.3
OVCAR-8	−45	−5.69	−4.36	–	−6.4
NCI/ADR-RES	66	−4.67	–	–	−6.4
SK-OV-3	81	–	–	–	−6.3
*Renal Cancer*					
786-0	41	−5.15	−4.25	–	−5.8
A498	44	−5.46	–	–	−4.6
ACHN	42	−5.16	–	–	−5.4
CAKI-1	−30	−5.63	−5.24	−4.33	−6.5
SN12C	43	−5.13	–	–	−5.1
TK-10	51	−4.98	–	–	−6.3
UO-31	−79	−5.88	−5.54	–	−6.1
RXF 393	−4	−5.62	−5.05	−4.42	−6.3
*Prostate C.*					
PC-3	11	−5.48	−4.84	−4.09	−5.5
DU-145	34	−5.33	−4.63	−4.09	−6.3
*Breast C.*					
MCF7	77	−4.19	–	–	−6.3
MDA-MB-231/ATCC	37	−5.20	–	–	ns
HS 578T	12	−5.48	−4.73	–	−5.2
BT-549	86	–	–	–	−5.9
T-47D	57	−4.77	–	–	−5.0
MDA-MB-468	20	−5.44	–	–	ns
MG_MID^e^		−5.1	−4.4	−4.09	

^a^Data obtained from the NCI’s in vitro disease-oriented human tumor cells

^b^Values greater than zero mean percentage of growth and those less than zero mean percentage of lethality to the tumor cell line

^c^The values greater than −4 were excluded

^d^Cell line not screened

^e^MG_MID (mean graph midpoint) arithmetical mean value for all tested cell lines

## Experimental

### Chemistry

Melting points were determined on a Boethius apparatus and were uncorrected. Elemental analyses for the synthesized compounds were performed on a Perkin Elmer 2400 (Waltham, MA, USA) analyzer, and results within ±0.4 % of the theoretical values were obtained for the new compounds. ^1^H-NMR and ^13^C-NMR spectra were acquired in *d*_6_-DMSO on a Bruker ARX 300 MHz (Bruker Analytic, Karlsruhe, Germany; Bruker AG, Fallanden, Switzerland) instrument. Tetramethylsilane was used as the internal standard and all chemical shift values were expressed in parts per million (δ, ppm). IR spectra were recorded on a Specord M80 spectrometer using KBr pellets. X-Ray Crystallography: the data were collected using the Bruker KAPPA APEXII ULTRA controlled by APEXII software. Reaction progress and the purity of the obtained compounds were monitored by thin-layer chromatography on Merck silica gel plates (Merck F_254,_ Darmstadt, Germany) using the solvent system dichloromethane: 1-propanol (10:1) for elution. Iodine was used as a developing agent. The chemicals and reagents for syntheses were obtained from Alfa Aesar (Karlsruhe, Germany), Chempur (Piekary Sl. Poland), and Sigma-Aldrich (Steinheim, Germany). Starting compounds are synthesized according to the literature (Gewald *et al*., 1966; Becan and Wagner, 2008).

#### General procedures for the synthesis of compounds **4a**–**4f** and **5a**–**5f**

To a solution of appropriate compound **2** or **3** (10 mmol) in acetonitrile (20 ml), diethyl sulfate (4.62 g, 30 mmol) was added, and the reaction mixture was heated under reflux for 1 h at 130 °C. After cooling, 100 ml of water was added and the reaction mixture was refluxed with stirring for 2 h during which the product was precipitated. The solid was filtered and suspended in a hot mixture of methanol and 5 % NaHCO_3_. The reaction mixture was allowed to cool, and the crude product was filtered and crystallized from appropriate solvent.

#### 3,5-Diphenyl-6*H*-thiazolo[4,5-*d*]pyrimidine-2,7-dione (**4a**)

IR (KBr) cm^−1^: 3450, 3080 (NH), 1680 (C=O), 1530 (C=N), 1260 (C–S–C), 760 (phenyl). ^1^H-NMR (*d*_6_-DMSO) δ: 7.42–7.93 (m, 10H, arom.), 13.19 (s, 1H, NH). Anal. Calcd for C_17_H_11_N_3_O_2_S: C, 63.54; H, 3.45; N, 13.08. Found: C, 63.44; H, 3.52; N, 13.27.

#### 5-(4-Chlorophenyl)-3-phenyl-6*H*-thiazolo[4,5-*d*]pyrimidine-2,7-dione (**4b**)

IR (KBr) cm^−1^: 3450, 3090 (NH), 1670 (C=O), 1590 (C=N), 1230 (C–S–C), 760 (phenyl). ^1^H-NMR (*d*_6-_DMSO) δ: 7.51–7.94 (m, 9H, arom.), 13.22 (s, 1H, NH). Anal. Calcd for C_17_H_10_ClN_3_O_2_S: C, 57.39; H, 2.83; N, 11.81. Found: C, 57.56; H, 3.01; N, 11.97.

#### 5-(2-Chlorophenyl)-3-phenyl-6*H*-thiazolo[4,5-*d*]pyrimidine-2,7-dione (**4c**)

IR (KBr) cm^−1^: 3470, 3080 (NH), 1680 (C=O), 1590 (C=N), 1260 (C–S–C), 760 (phenyl). ^1^H-NMR (*d*_6_-DMSO) δ: 7.34–7.99 (m, 9H, arom.), 13.27 (s, 1H, NH). Anal. Calcd for C_17_H_10_ClN_3_O_2_S: C, 57.39; H, 2.83; N, 11.81. Found: C, 57.59; H, 2.87; N, 11.85.

#### 5-(4-Fluorophenyl)-3-phenyl-6*H*-thiazolo[4,5-*d*]pyrimidine-2,7-dione (**4d**)

IR (KBr) cm^−1^: 3450, 3090 (NH), 1680 (C=O), 1610 (C=N), 1240 (C–S–C), 770 (phenyl). ^1^H-NMR (*d*_6_-DMSO) δ: 7.31–8.20 (m, 9H, arom.), 13.20 (s, 1H, NH). Anal. Calcd for C_17_H_10_FN_3_O_2_S: C, 60.17; H, 2.97; N, 12.38. Found: C, 59.98; H, 3.03; N, 12.41.

#### 3,5-Bis(4-fluorophenyl)-6H-thiazolo[4,5-*d*]pyrimidine-2,7-dione (**4e**)

IR (KBr) cm^−1^: 3470, 3090 (NH), 1690 (C=O), 1570 (C=N), 1240 (C–S–C), 780 (phenyl). ^1^H-NMR (*d*_6_-DMSO) δ: 7.22–8.03 (m, 8H, arom.), 13.21 (s, 1H, NH). Anal. Calcd for C_17_H_9_FN_3_O_2_S: C, 57.14; H, 2.54; N, 11.76. Found: C, 57.31; H, 2.55; N, 11.94.

#### 3-(4-Bromophenyl)-5-phenyl-6*H*-thiazolo[4,5-*d*]pyrimidine-2,7-dione (**4f**)

IR (KBr) cm^−1^: 3450, 3080 (NH), 1680 (C=O), 1590 (C=N), 1260 (C–S–C), 760 (phenyl). ^1^H-NMR (*d*_6_-DMSO) δ: 7.45–8.16 (m, 9H, arom.), 13.19 (s, 1H, NH). Anal. Calcd for C_17_H_10_BrN_3_O_2_S: C, 51.01; H, 2.52; N, 10.50. Found: C, 51.14; H, 2.60; N, 10.66.

#### 7-Chloro-3,5-diphenyl-thiazolo[4,5-*d*]pyrimidin-2-one (**5a**)

IR (KBr) cm^−1^: 1680 (C=O), 1590 (C=N), 1260 (C–S–C), 760 (phenyl). ^1^H-NMR (*d*_6_-DMSO) δ: 7.46–8.13 (m, 10H, arom.). Anal. Calcd for C_17_H_10_ClN_3_OS: C, 60.09; H, 2.97; N, 12.37. Found: C, 59.98; H, 3.01; N, 12.38.

#### 7-Chloro-5-(4-chlorophenyl)-3-phenyl-thiazolo[4,5-*d*]pyrimidin-2-one (**5b**)

IR (KBr) cm^−1^: 1680 (C=O), 1560 (C=N), 1230 (C–S–C), 760 (phenyl). ^1^H-NMR (*d*_6_-DMSO) δ: 7.52–8.11 (m, 9H, arom.). Anal. Calcd for C_17_H_9_Cl_2_N_3_OS: C, 54.56; H, 2.42; N, 11.23. Found: C, 54.60; H, 2.49; N, 11.29.

#### 7-Chloro-5-(2-chlorophenyl)-3-phenyl-thiazolo[4,5-*d*]pyrimidin-2-one (**5c**)

IR (KBr) cm^−1^: 1690 (C=O), 1570 (C=N), 1250 (C–S–C), 760 (phenyl). ^1^H-NMR (*d*_6_-DMSO) δ: 7.52–8.11 (m, 9H, arom.). Anal. Calcd for C_17_H_9_Cl_2_N_3_OS: C, 54.56; H, 2.42; N, 11.23. Found: C, 54.65; H, 2.50; N, 11.33.

#### 7-Chloro-5-(4-fluorophenyl)-3-phenyl-thiazolo[4,5-*d*]pyrimidin-2-one (**5d**)

IR (KBr) cm^−1^: 1690 (C=O), 1600 (C=N), 1240 (C–S–C), 760 (phenyl). ^1^H-NMR (*d*_6_-DMSO) δ: 7.27–8.15 (m, 9H, arom.). Anal. Calcd for C_17_H_9_ClN_3_OS: C, 57.10; H, 2.73; N, 11.75. Found: C, 57.21; H, 2.86; N, 11.83.

#### 7-Chloro-3,5-bis(4-fluorophenyl)thiazolo[4,5-*d*]pyrimidin-2-one (**5e**)

IR (KBr) cm^−1^: 1690 (C = O), 1590 (C = N), 1250 (C–S–C), 770 (phenyl). ^1^H-NMR (*d*_6_-DMSO) δ: 7.26–8.22 (m, 8H, arom.). Anal. Calcd for C_17_H_8_ClF_2_N_3_OS: C, 54.34; H, 2.15; N, 11.18. Found: C, 54.42; H, 2.20; N, 11.26.

#### 3-(4-Bromophenyl)-7-chloro-5-phenyl-thiazolo[4,5-*d*]pyrimidin-2-one (**5f**)

IR (KBr) cm^−1^: 1680 (C=O), 1560 (C=N), 1250 (C–S–C), 770 (phenyl). ^1^H-NMR (*d*_6_-DMSO) δ: 7.33–8.16 (m, 8H, arom.). Anal. Calcd for C_17_H_9_BrClN_3_OS: C, 48.77; H, 2.17; N, 10.04. Found: C, 48.91; H, 2.25; N, 10.12.

Characteristic data of the new compounds are depicted in Table 5.

**Table 5:** Characterization data of compounds **4a**–**4f** and **5a**–**5f**

Compound	R1	R2	m.p. (°C)	Yield %	Molecular formula	Molecular weight	Solvent
**4a**	–	–	279–280	64.55	C_17_H_11_N_3_O_2_ S	321,35	1-Propanol
**4b**	–	4-Cl	369–370	58.77	C_17_H_10_ClN_3_O_2_S	355,81	DMF-1-propanol 3:1
**4c**	–	2-Cl	338–339	58.88	C_17_H_10_ClN_3_O_2_S	355,81	DMF-1-propanol 3:1
**4d**	–	4-F	348–349	56.51	C_17_H_10_FN_3_O_2_S	339,31	DMF-water
**4e**	4-F	4-F	327–328	49.73	C_17_H_9_F_2_N_3_O_2_ S	357,33	1-Propanol
**4f**	4-Br	–	210–211	55.77	C_17_H_10_BrN_3_O_2_ S	400,25	DMF-1-propanol 3:1
**5a**	–	–	194–195	63.87	C_17_H_10_ClN_3_OS	339,80	DMF-1-propanol 3:1
**5b**	–	4-Cl	171–172	57.45	C_17_H_9_Cl_2_N_3_OS	374, 24	DMF-water
**5c**	–	2-Cl	240–241	52.32	C_17_ H_9_Cl_2_N_3_OS	374,24	DMF-1-propanol 3:1
**5d**	–	4-F	235–236	54.73	C_17_H_9_ClFN_3_OS	357,79	DMF-1-propanol 3:1
**5e**	4-F	4-F	234–235	48.67	C_17_H_8_ClF_2_N_3_OS	375,78	DMF-1-propanol 3:1
**5f**	4-Br	–	388–390	57.46	C_17_H_9_BrClN_3_OS	418,69	DMF-1-propanol 3:1

### Antitumor in vitro screening

The antitumor studies were performed at the NCI (Bethesda, MD, USA). The test agents were preliminarily evaluated in the full panel of 60 different human tumor cell lines derived from nine cancer diseases, namely leukemia, melanoma, non-small cell lung c., colon c., brain c., breast c., ovarian c., prostate c., and renal c., at single 10^−5^ M concentration. Compounds with significant GI are evaluated at five different concentrations ranging from 10^−4^ to 10^−8^ M. The percent growth was evaluated versus controls not treated with tested compounds. Preparation of the tested compounds and the sulforhodamine B (SRB) protein assay which was used to estimate cell viability of growth were described previously (Becan and Wagner, 2008; Monks *et al*., 1991; Boyd and Paull, 1995; Shoemaker *et al*., 2002).
